# Development and Characterization of Thermoformed Bilayer Trays of Paper and Renewable Succinic Acid Derived Biopolyester Blends and Their Application to Preserve Fresh Pasta

**DOI:** 10.3390/ma16103872

**Published:** 2023-05-21

**Authors:** Eva Hernández-García, Marta Pacheco-Romeralo, Pedro Zomeño, Gianluca Viscusi, Francesca Malvano, Giuliana Gorrasi, Sergio Torres-Giner

**Affiliations:** 1 Research Institute of Food Engineering for Development (IIAD), Universitat Politècnica de València (UPV), Camino de Vera s/n, 46022 Valencia, Spain; evherga1@upvnet.upv.es (E.H.-G.); mpacrom@upvnet.upv.es (M.P.-R.); 2 Packaging Technologies Department, AINIA, Calle Benjamín Franklin 5-11, 46980 Paterna, Spain; pzomeno@ainia.es; 3 Department of Industrial Engineering (DIIn), University of Salerno (UNISA), Via Giovanni Paolo II 132, 84084 Fisciano, Italy; gviscusi@unisa.it (G.V.); fmalvano@unisa.it (F.M.); ggorrasi@unisa.it (G.G.)

**Keywords:** paper, biopolymers, thermoforming, food preservation, migration

## Abstract

The present study reports on the development by thermoforming of highly sustainable trays based on a bilayer structure composed of paper substrate and a film made of a blend of partially bio-based poly(butylene succinate) (PBS) and poly(butylene succinate-*co*-adipate) (PBSA). The incorporation of the renewable succinic acid derived biopolyester blend film slightly improved the thermal resistance and tensile strength of paper, whereas its flexural ductility and puncture resistance were notably enhanced. Furthermore, in terms of barrier properties, the incorporation of this biopolymer blend film reduced the water and aroma vapor permeances of paper by two orders of magnitude, while it endowed the paper structure with intermediate oxygen barrier properties. The resultant thermoformed bilayer trays were, thereafter, originally applied to preserve non-thermally treated Italian artisanal fresh pasta, “fusilli calabresi” type, which was stored under refrigeration conditions for 3 weeks. Shelf-life evaluation showed that the application of the PBS–PBSA film on the paper substrate delayed color changes and mold growth for 1 week, as well as reduced drying of fresh pasta, resulting in acceptable physicochemical quality parameters within 9 days of storage. Lastly, overall migration studies performed with two food simulants demonstrated that the newly developed paper/PBS–PBSA trays are safe since these successfully comply with current legislation on plastic materials and articles intended to come into contact with food.

## 1. Introduction

The use of paper in food packaging applications has increased in the last few years due to the environmental and economic issues associated with plastic materials. In fact, most of the current plastics in the packaging industry rely on fossil derived polymers that are not biodegradable. Therefore, this makes them a great source of wastes, with nearly no value, posing a serious problem for the environment and the economy [1]. Furthermore, the lack of recycling of single-use plastics and their non-biodegradable characteristics facilitate their entry into natural ecosystems, leading to the formation of microplastics, which represents a growing concern [2,3]. In this context, paper is considered a more sustainable material than plastic, aluminum, or glass [4]. Paper can be defined as a material made from cellulose pulp that is obtained from ground vegetable fibers suspended in water, generally bleached, then dried and hardened [5]. It is a light material with a high mechanical resistance and opacity, and it is available in a wide range of grammages. Regarding its environmental impact, paper has two important advantages over the use of other materials for food packaging. On the one hand, it is based on a raw material of natural origin. On the other hand, it is biodegradable, even in certain environmental conditions. In particular, the disintegration time of paper and cardboard is only 1 year, in contrast to the 150 years it takes, on average, for a plastic film based on petrochemical polymers to disintegrate [6]. Furthermore, its recycling is substantially less complex than that of plastics, requiring less energy and fewer stages, which yields a high recyclability rate, of around 60–70%. In addition, the generation of paper and cardboard in its production process emits 70% lower carbon dioxide (CO_2_) emissions than plastics [7].

Despite the high environmental benefits of paper for packaging applications, it also has certain limitations when used as a monolayer material, which is particularly relevant for the preservation of fresh food with high water activity (a_w_). Paper is a hygroscopic material and, therefore, has low moisture resistance and high-water vapor permeability. In addition, it is highly permeable to organic vapors (aroma) and gases (oxygen) due to its high porosity as it consists of cellulose fibers [8]. Thus, paper packaging has barrier values that are totally insufficient to preserve moist foods or foods that can release water (meat, fish, fresh pasta, etc.). Furthermore, at high or moderate but prolonged temperatures, which are necessary for defrosting or cooking, paper can burn, especially at temperatures above 200 °C [9]. Moreover, in terms of mechanical properties, in order to withstand stress, paper is an excessively fragile material with low ductility. Given the fact that paper is not a heat-sealable material, its application as a single-layer material in packaging is restricted to foods with a short shelf-life or low barrier requirements, such as fruit and vegetables. These physical drawbacks, which are intrinsic to its characteristics, certainly limit the applications of paper in food packaging.

Therefore, the above characteristics of paper make it necessary to combine it, in most cases, with waxes, silicones, or thermoplastic materials [10]. For example, plastic coatings or external layers of low-density polyethylene (LDPE) are often incorporated onto the paper substrate to provide greater resistance to water and temperature. However, these materials significantly hinder the subsequent recyclability of the paper-based packaging, as well as the composting of paper [11]. In the context of the Circular Economy, the use of biopolymers, totally or partially derived from renewable sources and with the ability to biodegrade, can offer a more sustainable solution [12]. Biopolymers are macromolecules that are obtained or derived from biomass and can disintegrate in industrial compost or natural media [13]. Biopolymers that can be used as paper liner materials for food packaging include proteins (whey, wheat gluten, and zein), polysaccharides (chitosan, starch, lignocellulose derived compounds, and alginates), and, most relevantly, polyesters such as polyhydroxyalkanoates (PHAs) and polylactide (PLA). Among biopolyesters, the use of those derived from renewable succinic acid, such as polybutylene succinate (PBS) and poly(butylene succinate-*co*-adipate) (PBSA), or mixtures thereof, has notably increased in recent years [14].

Our previous study recently reported that double coatings of poly(3-hydroxybutyrate-*co*-3-hydroxyvalerate) (PHBV) can improve the barrier properties of paper to water vapor and gases, while having a minimal effect on its optical and thermal properties [15]. In addition, as these biopolyesters are thermoplastic materials, they can be effectively adhered to the paper substrate via a conventional heat-sealing process, resulting in packaging structures that offer the necessary properties in terms of performance and safety for food preservation applications. Thus, paper/biopolyester structures present themselves as excellent candidates for developing sustainable packaging for food preservation. However, the resultant water barrier and, more notably, mechanical performance of the double-coated PHBV paper sheets were still remarkably lower than those of equivalent multilayers developed by the same process using petrochemical polyethylene terephthalate (PET)-based films. Thus, other novel biopolymer/paper structures will have to be explored. In this regard, PBS is a biodegradable aliphatic semicrystalline polyester produced by polycondensation of succinic acid and 1,4-butanediol. Furthermore, succinic acid can be obtained through biological approach using microorganisms (*Anaerobiospirillum succiniciproducens*, *Actinobacillus succinogenes*, and *Mannheimia succiniciproducens*) [16] and renewable feedstock (e.g., sugar beets and wood residues) [17]. Nevertheless, PBS should be modified by blending or copolymerization with adipic acid to yield PBSA to improve its ductility and also disintegration rate to suit the required properties for food packaging [18]. Whereas PBS shows moderate rigidity and hardness, PBSA offers improved flexibility and impact strength. In this regard, the use of PBS and PBSA blends can offer higher mechanical performance, as well as facilitate the heat-sealing process with paper [19]. Furthermore, PBSA can facilitate the thermoformability of PBS to develop food packaging articles, such as trays, which can be particularly attractive for the preservation of foodstuffs with high a_w_ [20].

Therefore, the present study aims to resume previous research work dealing with multilayer structures of paper and biopolyesters. It originally shows the development and characterization of the physical performance of bilayer trays made of paper substrate and renewable succinic acid derived biopolyester blend films. To this end, the PBS–PBSA blends were first melt-mixed and cast-extruded into films, which were thereafter subjected, in combination with paper sheets, to a thermoforming process to produce bilayer trays. Then, the physical performance in terms of optical, thermal, mechanical, and barrier properties of the resultant bilayer paper/biopolyester trays were determined to ascertain their performance in food packaging applications. The properties of the newly developed paper/biopolyester structures were also compared with equivalent trays of monolayer paper and bilayer structures of paper with a high-barrier petrochemical polymer film. Subsequently, the resultant bilayer trays were originally applied to preserve locally produced fresh pasta. To this end, shelf life was analyzed and compared with the same fresh pasta packaged in trays made of monolayer paper and bilayer structures made of paper with a high-barrier petrochemical polymer film. Lastly, the food safety of the bilayer trays was analyzed by means of migration studies using food simulants.

## 2. Materials and Methods

### 2.1. Materials

Paper sheets with a grammage of 220 g/m^2^ and thickness of 290 µm were supplied by Bille-rudkornäs-CrownBoard Prestige™ (Solna, Sweden). This paper is derived from 100% wood fibers and is suitable for use in contact with foodstuffs (EU 10/2011 [21]). 

The biopolyester film, used as both the liner and the lid of the trays, was obtained from a mixture of PBS and PBSA biopolyesters, supplied by Mitsubishi Chemical Corporation (Tokyo, Japan) as BioPBS™ FZ91 PM and FD92 PM, respectively. Both grades are derived from renewable succinic acid, thus being partially bio-based polymers. Moreover, they are designed to be intended to come into contact with food since they comply with EU 10/2011 [21] and are also certified to be biodegradable under industrial (EN 13432 [22]), home (EN 17427 [23]), and soil (EN 13432 [22]) composting conditions.

A 100-µm multilayer film based on PET, an external layer of LDPE for sealing, and an inner layer of poly(ethylene-*co*-vinyl alcohol) (EVOH) as an oxygen barrier was supplied by Cryovac Inc. (Cryovac^®^ Darfresh^®^ VST300E TOP WEB, Sealed Air Spain, Buñol, Spain). This petrochemical multilayer, herein referred to as the PET film, was designed by the manufacturer for PET lidding trays in barrier packaging.

_D_-Limonene and poly(2,6-diphenyl-p-phenylene oxide) (MPPO) were purchased from Sigma-Aldrich S.A. (Madrid, Spain). Ethanol 96% and magnesium nitrate (Mg(NO_3_)_2_) were obtained from Panreac Química (Barcelona, Spain).

### 2.2. Development of Paper-Based Trays

The as-received PBS and PBSA pellets were first dried at 60 °C for 8 h in a vacuum oven (vacuum TEM-TJP, Selecta, S.A., Barcelona, Spain) to remove any residual water and avoid hydrolysis during thermal processing [24]. After this, the biopolyester pellets were weighed at a 1:2 PBS–PBSA (*w/w*) ratio and premixed manually in a zip bag. The pellets were then melt-mixed in a co-rotating ZSK-18 MEGAlab laboratory twin-screw extruder from Coperion (Stuttgart, Germany). This extruder has a diameter of 18 mm and a length-to-diameter (L/D) ratio of 48. Further details about the melt-mixing equipment can be found elsewhere [24]. The extrusion process was carried out at 25 rpm and with a temperature profile of 115–115–120–120–125–125–130 °C (from the hopper to the die) and the extruded materials were pelletized in an air-knife unit. The resultant pellets were cast-extruded using single-screw extruder in a TEACH-LINE^®^ E 20 T connected to a flat-film takeoff unit Chill-Roll 202 TEACH-LINE^®^ CR 72 T CR 72 T (Dr Collin GmbH, Ebersberg, Germany). The extruder is based on a single screw with a diameter (D) of 20 mm and length of 25 × D, having a flat die of 120 mm in width. The temperature profile was 35 °C (feeding)–115–120–120–120–130 °C (head) using a speed of 30 rpm. PBS–PBSA films of approximately 200 µm were attained by adjusting the speed of the calendar and the drag. Further details of the cast-roll machine and the calender flat-film system can be found elsewhere [25].

**Figure 1:**
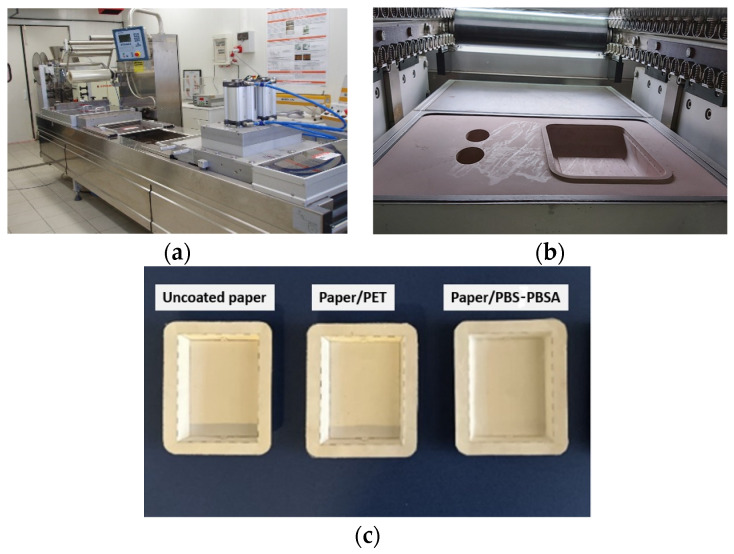
(**a**) Vacuum thermoformer; (**b**) mold chamber; (**c**) trays of paper, paper/polyethylene terephthalate (PET), and paper/poly(butylene succinate) (PBS) and poly(butylene succinate-*co*-adipate) (PBSA).

The resultant films were thereafter thermoformed with the paper sheets into bilayer trays of 120 mm × 80 mm (10 mm of upper sealing part) by means of a Multivac R-230 vacuum thermoformer (MULTIVAC UK, Wiltshire, UK) with a rated power of 11 kW. The optimal conditions for thermoforming were 105 °C and 8 bar for 12 s. The same thermoforming process was applied to obtain uncoated paper trays, as well as paper/PET trays (115 °C). Figure 1 shows the image of vacuum packaging equipment (Figure 1a) with details of the mold chamber (Figure 1b) and the resultant trays (Figure 1c).

### 2.3. Characterization of Paper-Based Trays

#### 2.3.1. Film Thickness and Conditioning 

The whole thickness of the film and sheet samples was determined at six random points using a digital electronic micrometer with an accuracy of ±0.001 mm (Palmer model COMECTA, Barcelona, Spain). To this end, the bottom of the bilayer trays was previously cut manually. The samples were thereafter conditioned at 25 °C and 53% relative humidity (RH) in desiccators containing Mg(NO_3_)_2_ for 1 week prior to characterization.

#### 2.3.2. Optical Evaluation

The optical properties were determined in triplicate by measuring the reflection spectrum of the samples from 360 to 700 nm of wavelength using a MINOLTA spectrocolorimeter (model CM-5, Minolta Co., Tokyo, Japan). The transparency was measured by means of the internal transmittance (T_i_), applying the Kubelka–Munk theory of the multiple dispersion of reflection spectrum given the reflection spectra of both black and white backgrounds. The CIE L*a*b* (CIELAB) color coordinates, chromatic parameters chroma (C_ab_*) and hue (h_ab_*), and total color difference (∆E_ab_*) were obtained by considering illuminant D65 and observer 10° from the reflectance of an infinitely thick layer of the material using Equations (1)–(6) [26,27].

(1) $${\;T}_{i}=\sqrt{{\left(a+R_{0}\right)}^{2}-b^{2}}.$$

(2) $$a=\frac{1}{2}\;\left[R+\left(\frac{R_{0}-R+R_{g}}{R_{0}\times R_{g}}\right)\right].$$

(3) $$b=\sqrt{a^{2}-1}.$$

(4) $$h_{\mathrm{ab}}{}^{*}=\;\mathrm{arctg}\left(\frac{b^{*}}{a^{*}}\right).$$

(5) $$C_{\mathrm{ab}}{}^{*}=\sqrt{a^{*2}+b^{*2}}.$$

(6) $${\Delta E}_{\mathrm{ab}}{}^{*}=\sqrt{{\left({\Delta L}^{*}\right)}^{2}+{\left({\Delta a}^{*}\right)}^{2}+{\left({\Delta b}^{*}\right)}^{2}}.$$

#### 2.3.3. Microstructural Analysis

The cross-sections of the monolayers and bilayers were observed by field-emission scanning electron microscopy (FESEM) in a JEOL model JSM-5410 (Tokyo, Japan). The samples were cryo-fractured in liquid nitrogen, mounted on the observation holders using double-sided carbon tape, and covered with a platinum layer (EM MED020 sputter coater, Leica Biosystems, Barcelona, Spain). An acceleration voltage of 2.0 kV was used, and the layer thicknesses were determined using the ImageJ v1.53c Program.

#### 2.3.4. Thermal Characterization

Thermal stability was determined by thermogravimetric analysis (TGA) in a TGA 1 STAR^e^ System analyzer (Mettler-Toledo, Greifensee, Switzerland). For the measurements, approximately 3–4 mg of each sample was analyzed in air atmosphere with a constant flow-rate of 10 mL/min. The heating program was carried out from 25 °C to 700 °C at a heating rate of 10 °C/min. The thermogravimetric and derivative curves were analyzed using a STAR^e^ Evaluation Software (Mettler-Toledo, Greifensee, Switzerland) to obtain the onset degradation temperature (temperature corresponding to the beginning of mass loss), the degradation temperature (T_deg_) that was obtained from the maximum value of the first derivative, and the remaining mass at 700 °C.

#### 2.3.5. Mechanical Analysis 

The mechanical behavior of the film and sheet samples was analyzed using a universal testing machine (Stable Micro System TA-XT plus, Haslemere, UK). Tensile tests were performed following ASTM standard method D882 [28], and the samples were mounted in the extension grip and stretched at a rate of 50 mm/min until breaking. Force–distance curves were obtained and transformed into stress–strain curves by considering sample dimensions and degree of deformation. Tensile tests were also performed on both the trays and lid films after 21 days of storage under the controlled temperature and humidity of 5 ± 1 °C and 85% ± 5% RH (Ja Car, VDM Refrigeration, Portici, Italy), which simulate storage conditions of the trays in the fridge. Similarly, flexural tests were performed on the paper-based sheet samples according to ISO 178 [29], where the speed rate was 50 mm/min. For both tensile and flexural tests, eight preconditioned samples of each formulation with dimensions of 25 mm × 100 mm were used.

The seal strength of the bilayer trays was determined in the universal testing machine following the previously developed methodology [30]. Briefly, the films were heat-sealed on one edge of the paper substrate in a hydraulic press (Model LP20, Labtech Engineering, Bangpoo, Thailand) and protected using Teflon sheets [15]. Sheet strips sizing 7.62 cm × 2.54 cm were sealed in a 2 × 2.54 cm^2^ area. The resultant bilayer samples were then stored at 25 °C and 53% RH for 1 week. Evaluation of the paper/film adhesion was performed according to ASTM F88/F88M-15 [31], using ten replicates. To do this, the unsealed edges of the samples (paper and film) were attached to the grips, separated by 50 mm. Thereafter, the samples were stretched at 200 mm/min, and the sealing strength was determined from the average force calculated in 80% of the total force–distance curve, as described in the standard method, according to Equation (7).

(7) $$\mathrm{Seal}\;\mathrm{strength}=\frac{\mathrm{Mean}\;\mathrm{Force}\;\left(N\right)}{\mathrm{Film}\;\mathrm{width}\;\left(m\right)}.$$

Puncture resistance was also determined for the paper-based trays in the universal testing machine, as described in ASTM F1306 [32]. To this end, a penetration probe of 2 mm moved toward the outer side of clamped sheets sizing 40 mm × 120 mm with a speed of 50 mm/min until the samples were penetrated. Maximum force (F_max_), total displacement (d_total_), and total energy (E_puncture_) were determined in quintuplicate.

#### 2.3.6. Permeance Measurements

Water vapor permeance was determined gravimetrically, at 25 °C and an RH gradient of 53–100%, following a modification of the ASTM E96-96M gravimetric method [33]. Payne permeability cups with the samples were weighed periodically using an analytical balance (ME36S, Sartorius, Göttingen, Germany, ±0.00001 g) at intervals of 1.5 h for 24 h after the steady state was reached. The water vapor permeance was calculated from the water vapor transmission rate (WVTR), determined from the slope of the weight loss versus time, and corrected for permeant partial pressure. For limonene vapor permeance, the procedure was similar to that described for water vapor with the difference that 5 mL of _D_-limonene was placed inside the Payne permeability cups, which were stored under the controlled room conditions of 25 °C and 53% RH [34]. In both cases, cups with aluminum films were used as control samples to estimate and subtract the vapor loss through the sealing. Furthermore, films without water and _D_-limonene were used to correct the mass corresponding to the vapor gained in the film samples during analysis. For the monolayers, permeance was corrected for sample thickness to obtain permeability. All the vapor permeability measurements were performed in triplicate.

Oxygen permeance was determined by following the ASTM standard method D3985-05 [35]. Three film replicates of 50 cm^2^ of each formulation were measured using the Ox-Tran equipment (Model 1/50, Mocon, Minneapolis, MN, USA) at 25 °C and 53% RH. Permeance was calculated by dividing the oxygen transmission rate (OTR) by the difference in oxygen partial pressure between the two sides of the film. Similarly, in the case of the monolayers, permeance was corrected for sample thickness to obtain permeability. Measurements were recorded in triplicate.

### 2.4. Shelf-Life Evaluation of Fresh Pasta

#### 2.4.1. Packaging and Storage

**Figure 2:**
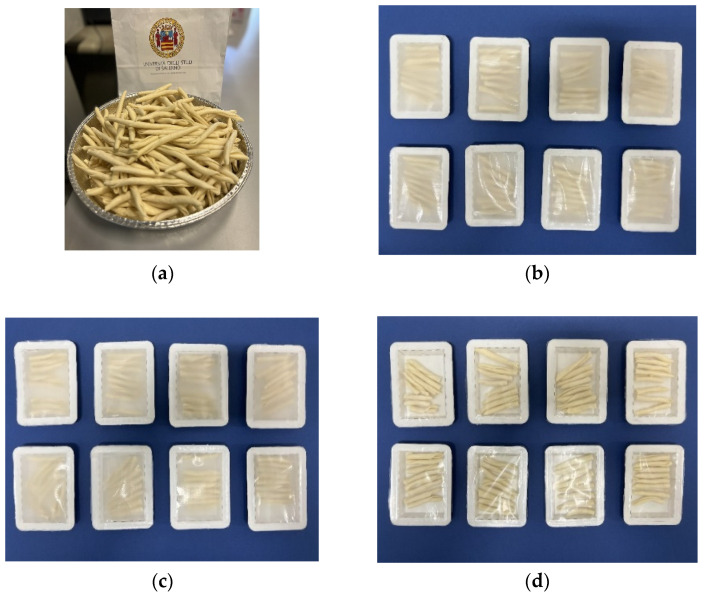
(**a**) As-received fresh pasta. Fresh pasta packaged in trays of: (**b**) uncoated paper, (**c**) paper/poly(butylene succinate) (PBS) and poly(butylene succinate-*co*-adipate) (PBSA), and (**d**) paper/polyethylene terephthalate (PET).

Locally produced traditional fresh durum wheat semolina “fusilli calabresi” type pasta (La Pasteria SNC, Salerno, Italy) was used to evaluate the performance of the newly developed trays to preserve food. For this purpose, 12 ± 1 g of the as-received pasta was added to the trays, which were all handled under aseptic conditions inside a cabinet (LOGIKA 120, BICASSA, Bernareggio, Italy), previously sterilized with ultraviolet (UV) light for 30 min. The same films used for the bilayer structures were applied as the lids in the trays. In the case of the uncoated paper tray, the PBS–PBSA blend film was used as lid. After the incorporation of the fresh pasta, the lid films were heat-sealed onto the trays using a heat impulse sealing equipment (FS-700H Hualian PLASTIC FILM SEALER, Zhejiang, China). Right after the packaging, the trays containing the fresh pasta were placed in the Ja Car chamber under the controlled temperature and humidity conditions of 5 ± 1 °C and 85% ± 5% RH. Figure 2 shows the as-received fresh pasta (Figure 2a) and the prepared food trays containing the pasta prior to analysis (Figure 2b–d). Fresh pasta without packaging was also used as control.

#### 2.4.2. Evaluation of Shelf-Life

Shelf-life characterization of the fresh pasta was carried out during a whole storage period of 3 weeks. Evaluation consisted of the visual observation of the fresh pasta packaged in the trays and the quantification of color properties using the MINOLTA spectrocolorimeter (model CM-5). Gravimetric measurements were also performed by weighing the food samples in the analytical balance (ME36S, Sartorius). The moisture content of the pasta was also determined, in triplicate, by drying in an oven (vacuum TEM-TJP) at 105 °C until achieving stable weight. Lastly, a_w_ was determined using a Testo 650 Humidity Meter (Testo Spa, Séttimo Milanese, Italy) and an electronic hygrometer with an accuracy of ±0.01. All tests were performed in triplicate at the following storage times: 4, 9, 13, 17, and 21 days.

### 2.5. Overall Migration Tests

Overall migration of the PBS–PBSA film, paper sheet, and their bilayer trays was evaluated using two food simulants, namely 10% *v*/*v* ethanol (simulant A), according to UNE-EN 1186-5 standard [36], and simulant Tenax (simulant E), following the UNE-EN 14338 standard [37]. Whereas simulant A is assigned to foods with a hydrophilic character, simulant E corresponds to poly(2,6-diphenyl-p-phenylene oxide) (MPPO, Sigma-Aldrich, S.A., Madrid, Spain). The latter is used to perform migration tests for dry or solid foods, but it is habitually used to determine the migration characteristics of paper due to its poor water resistance. Both tests were performed in triplicate by direct contact, without immersion, of the film or sheet sample with the food simulants at the normalized conditions of 40 °C for 10 days. The ratios of contact surface area to mass of food simulant were 20 dm^2^/kg for simulant A and 1 dm^2^/4 g for Tenax.

### 2.6. Statistical Analysis 

Results were subjected to analysis of variance (ANOVA) using Statgraphics Centurion XVII-64 software (Manugistics Corp., Rockville, MD, USA). Significant differences were assumed with a significance level greater than 95% (*p* < 0.05). 

## 3. Results

### 3.1. Optical Properties

**Figure 3:**
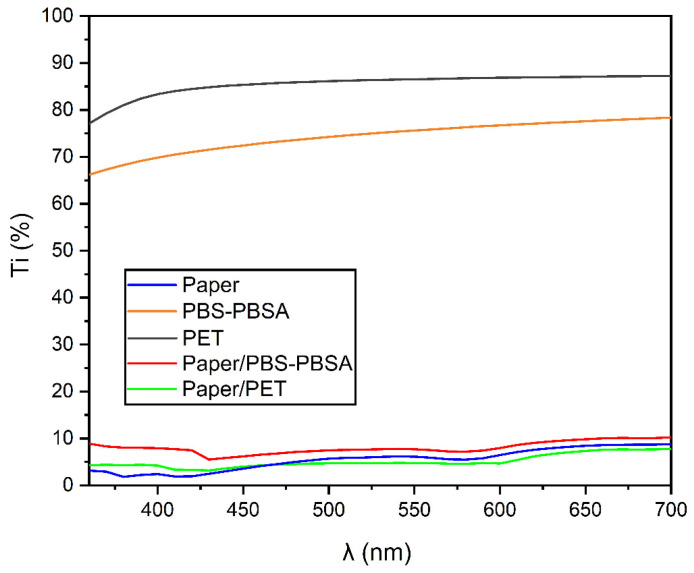
Spectral distribution curves of the percentage internal transmittance (T_i_) of the paper sheet, poly(butylene succinate) and poly(butylene succinate-*co*-adipate) (PBS–PBSA) blend and polyethylene terephthalate (PET) films, and paper/PBS–PBSA and paper/PET trays.

Figure 3 shows the spectral distribution curves, which represent the internal transmittance (T_i_) of the sheet, film, and tray samples as a function of the wavelength (λ). It can be seen that the paper-based samples were opaque since these sheets showed low T_i_ values, which are related to low light transmittance. In this regard, the different refractive indices of cellulose and air (1.5 and 1.0, respectively) cause the light scattering at the fiber surfaces and manifest as the opacity of paper [38]. Moreover, the opacity of paper is habitually increased by the addition of titanium dioxide (TiO_2_), and it is also affected by the level of pulp hydration and grammage of the paper [4]. Similar results were also observed for paper double-coated with PHBV films [15]. Thus, the paper-based trays had a very low transmittance percentage of light, which may be advantageous for protection against the oxidative processes of certain foods, such as oils or meat products [39]. In this regard, the micrometer-sized fibers of paper also partly reduced the light passage transmittance of the ultraviolet type A (UVA) region, seen below 400 nm. However, one should consider that, under UV light, the C−C bonds in the paper fiber structure will break, which can lead to a notable decrease in its mechanical properties [40].

Table 1 summarizes the results of the optical evaluation carried out on the film, sheet, and tray samples. It can be observed that the paper sheet showed the highest value of luminosity, whereas both the biopolyester blend and the PET films presented similar luminosities, being significantly higher (*p* < 0.05) in the case of the petrochemical polymer. For the bilayer paper/PBS–PBSA structures, used in the trays, the incorporation of the biopolyester blend film significantly reduced (*p* < 0.05) the luminosity of paper. However, this was not significantly different (*p* > 0.05) in the case of the paper/PET trays. The latter effect can be ascribed to lower luminosity of the biopolyester film since it is based on a blend of biopolymers with different refraction indices [41]. Furthermore, both PBS–PBSA and PET films showed values of −0.44 and 1.44 (a*) and −1.54 and 4.25 (b*), respectively, while the paper sheet presented values of approximately a* = 1.56 and b* = −5.83. In terms of the chroma (C_ab_*), values of 6.03, 1.52, and 4.52 were observed for the paper sheet, PBS–PBSA, and PET films, respectively, while the bilayer paper/PBS–PBSA and paper/PET trays showed values equal to 0.63 and 3.58, all being significantly different (*p* < 0.05). The hue or shade (h_ab_*) of the PBS–PBSA and PET films was located in the 100–110 range, corresponding to a slight yellow-to-orange hue, whereas the paper and bilayer trays had a similar angle, in the 280–290 range, which corresponds to a blue hue. This result agrees with our previous findings for paper double-coated with PHBV and PET films, which exhibited more bluish (lower h_ab_* values) but slightly less saturated (lower C_ab_* values) color in comparison with uncoated paper [15]. Lastly, the color difference (∆E_ab_*) values between the paper sheet and the bilayer trays of paper/PBS–PBSA and paper/PET were 5.42 and 2.48, respectively. This suggests that the optical properties of the paper were modified, particularly for the paper/PBS–PBSA bilayer structure, where ∆E*_ab_ > 5, thus an unexperienced observer would notice different colors [42]. The high color difference attained in the biopolymer-containing structure can be related to the use of a biopolymer blend that generally results in a hazy film with low transparency [43].

**Figure 4:**
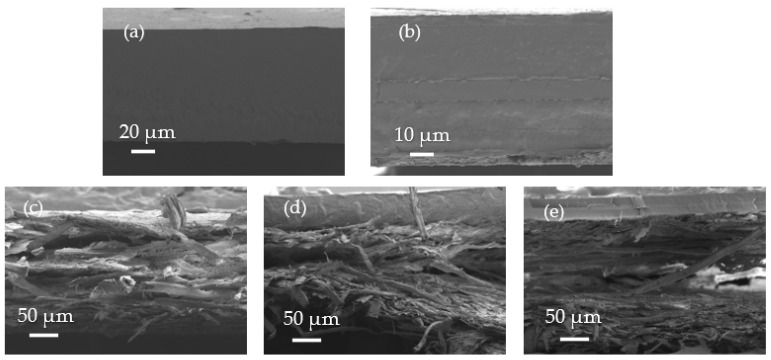
Field-emission electron microscopy (FESEM) micrographs taken at the cross-section of the poly(butylene succinate) and poly(butylene succinate-*co*-adipate) (PBS–PBSA) blend film (**a**), polyethylene terephthalate (PET) film (**b**), paper sheet (**c**), paper/PBS–PBSA tray (**d**), and paper/PET tray (**e**).

Figure 4 shows the cross-sectional morphologies of the polyester films, paper sheet, and bilayer tray samples obtained by FESEM. One can observe in Figure 4a,b that the PBS–PBSA and PET films showed a continuous section characteristic of a plastic material. Whereas the PBS–PBSA film presented a continuous structure that correspond to a single layer, the petrochemical film was composed of several layers, where the thickest layers, shown on both sides, would correspond to the polyester. Similar morphologies, based on multilayer structures containing EVOH inner layers, have been reported for high-barrier films [34]. In contrast, Figure 4c shows that the uncoated paper sheet presented an average thickness of approximately 290 µm, and it was composed of micrometer-sized fibers with an average diameter of nearly 20 µm [15]. In the case of the paper/PBS–PBSA and paper/PET trays, shown in Figure 4d,e, it was confirmed that their cross-sectional morphologies were based on a bilayer structure. Furthermore, both were seen to present good adhesion between the polyester layers and paper substrate, suggesting adequate mechanical resistance for handling and transport in food packaging.

### 3.2. Thermal Properties 

**Figure 5:**
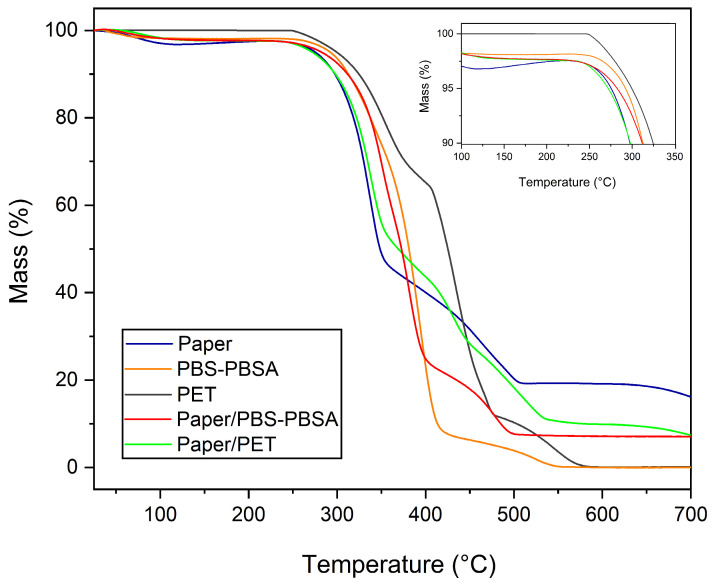
Thermogravimetric analysis (TGA) curves of the paper sheet, poly(butylene succinate) and poly(butylene succinate-*co*-adipate) (PBS–PBSA) blend and polyethylene terephthalate (PET) films, and paper/PBS–PBSA and paper/PET trays.

Figure 5 gathers the TGA curves of all developed samples, that is, the paper sheet, PBS–PBSA and PET films, and paper/PBS–BSA and paper/PET trays. From these curves, the corresponding values of T_onset_ (temperature of initial degradation), T_deg_ (temperature at maximum degradation rate), and residual mass at 700 °C were determined, and the results are included in Table 2. One can observe that the paper sample was thermally stable up to approximately 280 °C, while the thermal stability of the biopolyester blend and PET films was slightly higher (~300 °C). This result agrees with the thermal stability previously reported for paper, showing that decomposition occurs between 220 and 390 °C [40]. Thus, the thermal stability of paper was improved in both bilayer structures used to form the trays, increasing the thermal resistance by approximately 5–10 °C. In this context, Seoane et al. [44] reported similar results for paperboard/poly(3-hydroxybutyrate) (PHB) structures that were prepared by compression molding, showing that thermal degradation of paper occurs at 280 °C. Therefore, both thermoplastic layers offered to paper a slight improvement in thermal stability, and the bilayer trays were able to withstand temperatures close to ~290 °C. Thus, they can be adequate for most packaging applications that do not make use of temperatures above 250 °C, such as microwave heating or low-temperature cooking in oven [4].

In terms of the thermal degradation profile of the trays, three main mass losses were seen to occur in the samples. These took place at temperatures of approximately 100 *°*C, 335 *°*C, and 472 *°*C, which have been described for lignocellulosic materials [45]. Briefly, the first mass loss is related to moisture evaporation. The second one, which took place in the 310–350 °C range, is referred to as the “active pyrolysis zone” since the mass loss rate is high. The third one, seen to occur from 350 °C to above 520 °C, represents the “passive pyrolysis zone” since the mass loss rate is much lower. The second mass loss corresponds to the decomposition of hemicellulose and cellulose, whereas the third mass loss is ascribed to the lignin thermal degradation [46]. Moreover, the paper sample showed a remaining mass of approximately 16% at 700 *°*C that corresponds to inorganic materials and ashes generated from the organic material decomposition in an inert atmosphere. In the case of the polyester films, an additional thermal loss in the 500–600 *°*C range was observed, which has been ascribed to the thermal decomposition of the organic mass produced during the previous steps [47]. This thermal degradation step was not seen in the case of the paper-based samples since it overlapped with the decomposition of lignin.

### 3.3. Mechanical Properties

**Figure 6:**
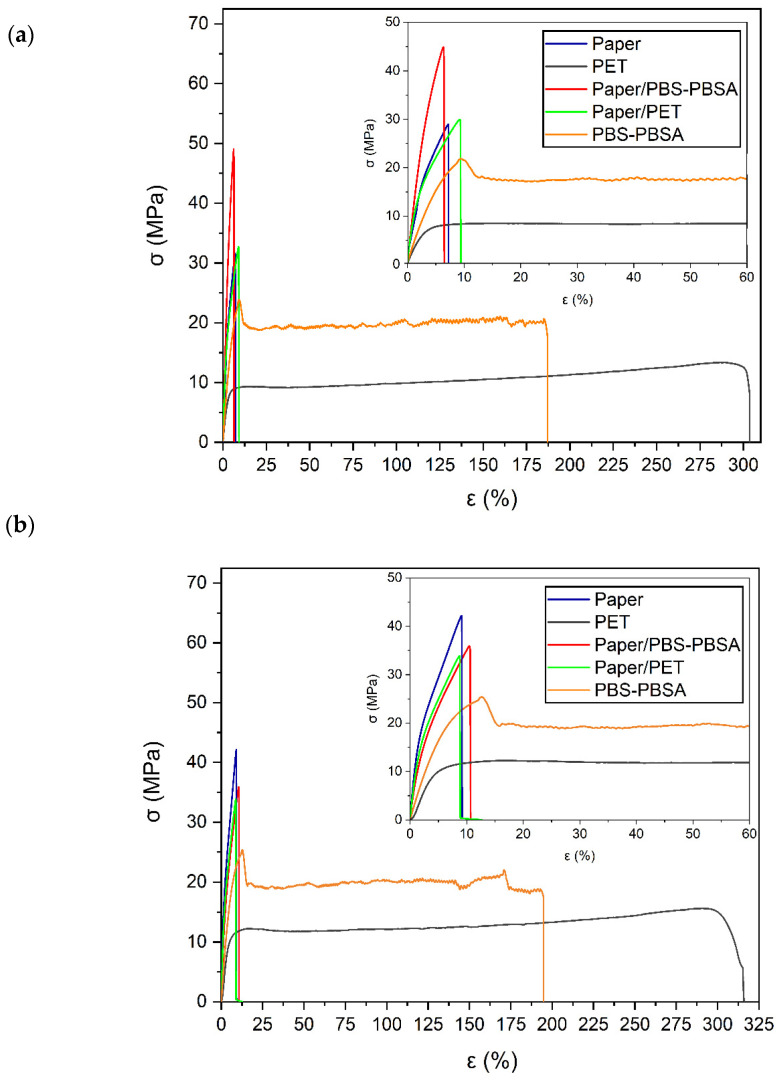
Typical tensile stress (σ) vs. deformation (ε) curves of the paper sheet, poly(butylene succinate) and poly(butylene succinate-*co*-adipate) (PBS–PBSA) blend and polyethylene terephthalate (PET) films, and paper/PBS–PBSA and paper/PET trays: (**a**) prior to storage; (**b**) after 21 days of storage at 5 °C and 85% relative humidity (RH).

Figure 6 shows the tensile stress–strain curves of the paper sheet, PBS–PBSA and PET films, and paper/PBS–BSA paper/PET trays. These curves allowed obtaining the mechanical parameters of tensile modulus (E_tensile_), tensile strength at yield (σ_y tensile_), and deformation at break (ε_b_), which are presented in Table 3. Figure 6a shows the mechanical curves of the samples prior to storage. On the basis of the presented results, paper can be considered a brittle and rigid material, characterized by high E_tensile_ (1787 MPa) and σ_y tensile_ (31.9 MPa) values, but with low ductility (ε_b_ ≈ 7%). In this sense, the mechanical performance of paper is dependent on the strength of its cellulose fibers, their surface area, length, and bonding strength [48]. Both monolayer films of PBS–PBSA and PET presented significantly lower (*p* < 0.05) values of E_tensile_ and σ_y tensile_, but broke after deformations of approximately 150% and 300%, respectively. The mechanical properties of the bilayer trays were still in the range of those of the monolayer paper, with moderate improvements in the mechanical resistance and no significant differences (*p* > 0.05) in ductility. In this regard, a slight mechanical enhancement in terms of ductility was also observed by Zhu et al. [38], where the elongation of paper increased from 1.65% to 2.75% after coating with cross-linked copolymers of chitosan and tannin extract-based epoxy. Similarly, other previous studies have demonstrated that the mechanical properties of Kraft paper coated with biopolymers, such as chitosan or starch, are still controlled by the cellulose fiber matrix, having a slight decrease in mechanical strength and increase in ductility [49,50,51,52].

Figure 6b shows the mechanical curves obtained after keeping the samples for 21 days in refrigeration conditions (5 *°*C and 85% RH). Interestingly, one can further observe that the paper-based samples presented a dissimilar performance after storage for 21 days. Thus, the neat paper sheet showed a significant reduction (*p* < 0.05) in E_tensile_, reaching a value of 1124 MPa, whereas the sample was slightly more ductile (ε_b_ ≈ 9%). This reduction in terms of elasticity, by approximately 37% when compared with the dry paper, can be ascribed to the plasticizing effect of water during storage. However, both polyester films presented similar mechanical parameters, showing no significant differences (*p* > 0.05) due to storage time. Nevertheless, both bilayers presented lower mechanical performance after storage due to the presence of paper. For the paper/PBS–PBSA tray, E_tensile_ was reduced from 1081 MPa to 859 MPa, which represents a percentage reduction of nearly 20%. In the case of the paper/PET tray, it was also reduced from 946 MPa to 924 MPa, that is, 2–3%, showing no significant differences (*p* > 0.05). Therefore, the paper substrate was effectively protected from moisture by the films. However, in the case of the biopolymer blend film, the mechanical performance was slightly impaired during storage in humid conditions. Furthermore, both resultant trays would still be restricted to rigid applications that can withstand certain stresses but do not require high deformations.

**Figure 7:**
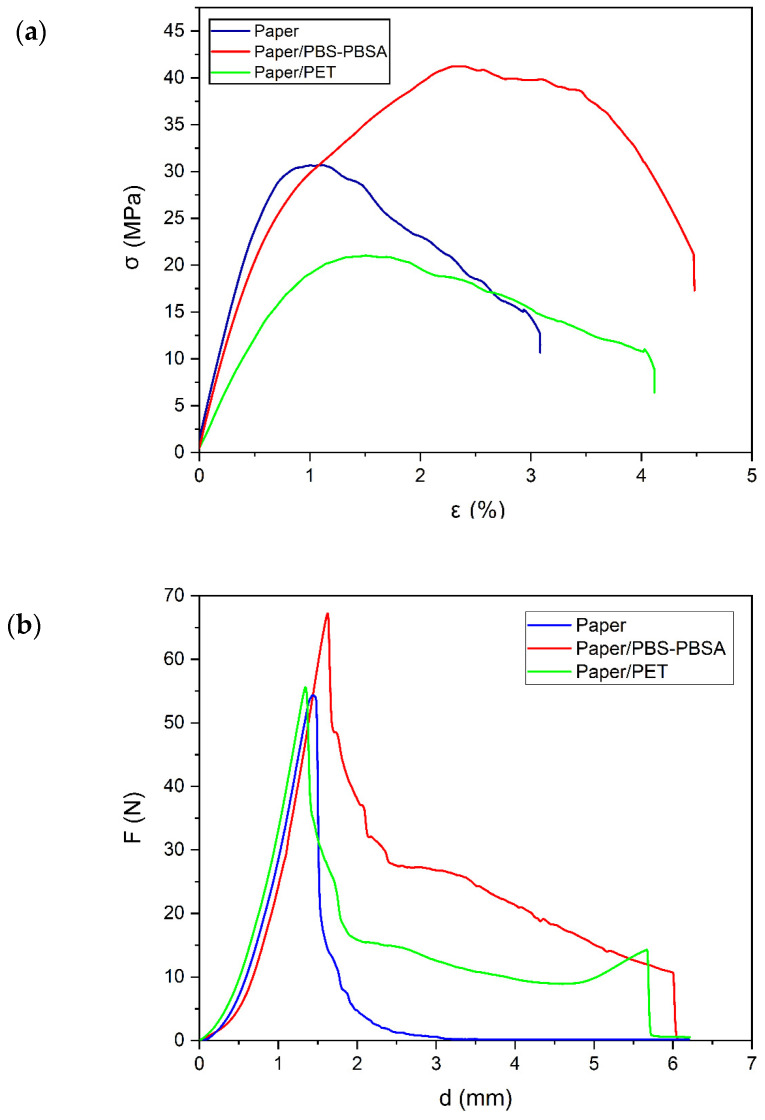
(**a**) Typical flexural stress (σ) vs. deformation (ε) curves and (**b**) force (F) vs. displacement (d) during the puncture process of the paper sheet, poly(butylene succinate) and poly(butylene succinate-*co*-adipate) (PBS–PBSA) blend and polyethylene terephthalate (PET) films, and paper/PBS–PBSA and paper/PET trays.

The effect of the polyester films on the mechanical properties of the paper substrate was also evaluated by means of flexural and puncture tests. The curves of these mechanical analyses are included in Figure 7. In this regard, flexural properties play an important role in defining the performance of paper packaging materials. In particular, they define the tendency of a material to bend, and one of the main advantages of paper is its high bending stiffness in relation to its relatively low weight. Figure 7a shows the flexural curves of the trays of paper, paper/PBS–PBSA, and paper/PET. Moreover, Table 4 provides the values of flexural modulus (E_flexural_), flexural strength at yield (σ_y flexural_), and elongation at yield (ε_y flexural_) obtained from these curves. Thus, one can observe that the mechanical performance of paper was notably modified, in a similar way previouly observed during the analysis of the flexural properties. Thus, for neat paper, E_flexural_, also known as the bending modulus, was significantly (*p* < 0.05) reduced by the incorporation of the polyester films. In particular, E_flexural_ decreased from nearly 1500 MPa, for the neat paper tray, to 1235 MPa and 912 MPa, for the paper/PBS–PBSA and paper/PET trays, respectively. Interestingly, the presence of the PBS–PBSA blend film increased σ_y flexural_, from 30.4 MPa to 35.9 MPa, as well as ε_y flexural_, from 0.69% to 1.14%. This improvement in the bending response of paper can be ascribed to the combination of the inherently high strength of PBS and high flexibility of PBSA, which show values of E_flexural_ and σ_y flexural_ for the homo- and copolyesters of 500–600 MPa and 30–40 MPa [53,54] and 300–450 MPa and 10–20 MPa [55,56], respectively. In the case of the paper/PET tray, this sample was able to deform to a higher extend, reaching a value of ε_y flexural_ of 1.36%, but the σ_y flexural_ was lower than in the other paper samples, that is, 20.3 MPa. Furthermore, the mechanical improvement attained in the paper trays with the incorporation of the biopolyester blend was confirmed by puncture resistance tests. Figure 7b shows the puncture curves for the paper, paper/PBS–PBSA, and paper/PET trays. The puncture results showed that the paper tray presented moderate force but low displacement and energy. Interestingly, the values of puncture resistance force and energy of the paper/PBS–PBSA trays were high, and displacement was moderate. Therefore, F_max_, d_total_, and E_puncture_ respectively increased from 53 MPa, 1.4 mm, and 25 mJ, for the neat paper tray, to 64 MPa, 5.4 mm, and 130 mJ, for the the paper/PBS–PBSA tray. Moreover, a moderate puncture force and energy with a high displacement in the puncture test of the paper/PET trays was achieved. Thus, the energy was significantly lower (*p* < 0.05) when compared to the bilayer trays of paper and renewable succinic acid derived biopolyester blends. This can be ascribed to the fact that the PBS–PBSA blends are mechanically strong and tough at the same time [57]. For instance, a value of E_puncture_ as high as 194 mJ was reported for extruded PBS films, however, caution must be taken in the comparison of the puncture resistance results because of the different thickness among the different samples [58].

**Figure 8:**
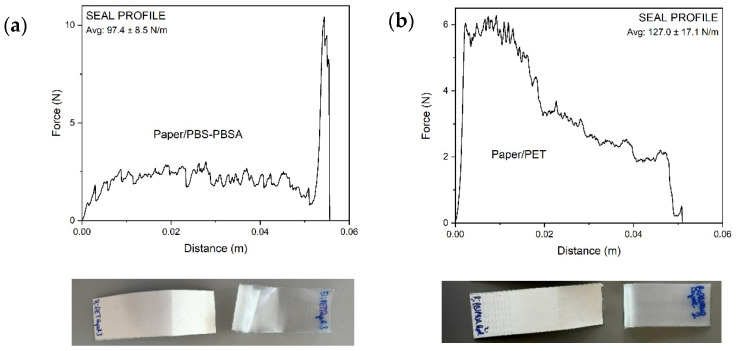
Typical force vs. distance curves of the paper/poly(butylene succinate) (PBS) and poly(butylene succinate-*co*-adipate) (PBSA) trays (**a**) and paper/polyethylene terephthalate (PET) trays (**b**) with mean values of seal strength (N/m) and images of the bilayer separation by delamination.

Thermo-sealing of polymer films on paper substrate is a complex process that is influenced by different factors, e.g., polymer–paper compatibility, interfacial energy between both surfaces, and structural changes occurring during storage [59]. Thus, the analysis of the seal strength in the bilayer structures is of relevance to ensure the material’s functionality in food packaging. In this test, the bilayer samples were unsealed at the edges to produce the peel arms, where one edge was paper and the other edge was film. Then, the peel arms were clamped in the grips of the tensile tester and pulled apart at a constant speed. Figure 8 shows the force–distance curves of the bilayer structures of paper/PBS–PBSA (Figure 8a) and paper/PET (Figure 8b). The images included below the mechanical curves are representative pictures of the bilayer samples obtained after the mechanical test. As can be seen in the Videos S1 and S2 (see Supplementary Materials), both test strips peeled apart in the seal area. Therefore, both bilayer structures exhibited delamination detachment rather than cohesive failure, breaking, tearing, or elongation of the substrate paper. The average peel force, measured by the testing machine as a part of the test cycle, was also gathered in the graphs, showing values of 97.4 and 127 N/m for the paper/PBS–PBSA and paper/PET samples, respectively. In general terms, although both bilayer structures presented delamination, they exhibited high sealing, particularly in the case of the petrochemical film, thus indicating that strong adhesion forces were established between the paper substrates and polyesters. Adhesion tests were similarly carried out by Seoane et al. [44] on paperboard/PHB structures. The authors showed that the PHB-based layer was also peeled off with torn paperboard fibers, up to the final failure of the PHB layer, suggesting that the interfacial adhesion also presented greater resistance to tear.

### 3.4. Barrier Properties

Table 5 gathers the permeance and permeability values of water and limonene vapors and oxygen gas of the paper sheet, PBS–PBSA and PET films, and paper/PBS–PBSA paper/PET trays. The barrier performance to these vapors and gases is, in fact, of main interest for food packaging. The barrier properties were expressed in terms of permeance since it represents the actual amount (mass or volume) of permeant per unit of time, area, and difference of partial pressure passing through a multilayer structure that is formed by materials of different permeabilities at the tested temperature and %RH conditions. In the case of the monolayers, that is, the paper substrate and PBS–PBSA and PET films, the permeability was also determined by correcting permeance with the thickness sample.

Water vapor barrier is of great importance for shelf-life extension since most of the physical and chemical deteriorations are related to equilibrium moisture content [60]. This is of high relevance in the case of paper packaging since it is composed of hygroscopic cellulose fibers forming a network with high porosity. As reported in Table 5, the water vapor barrier of the paper sheet is very limited with a permeance value of 1.10 × 10^−8^ kg/m^2^·Pa·s, resulting in a permeability value of 3.21 × 10^−12^ kg·m/m^2^·Pa·s, which agrees with the value reported previously [15]. In contrast, the biopolyester blend film and, more notably, the PET film presented significantly lower (*p* < 0.05) permeability values to water vapor. In particular, the 200-µm PBS–PBSA film resulted in a permeance of 1.47 × 10^−10^ kg/m^2^·Pa·s, yielding a permeability of 3.16 × 10^−14^ kg·m/m^2^·Pa·s. This barrier performance is in the range of the permeability reported for other biopolyesters, such as PHBV, PLA, or PBAT, which are adequate for medium-water-barrier packaging [24]. In contrast, the 100-µm PET film yielded a permeance value of 5.50 × 10^−11^ kg/m^2^·Pa·s, which corresponds to a permeability of 5.58 × 10^−15^ kg·m/m^2^·Pa·s, considering it as a monolayer material. This water vapor permeability is one order of magniture lower than that of the biopolyester blend since it is based on a multilayer structure containing polyolefins, such as LDPE (1.2 × 10^−15^ kg·m/m^2^·Pa·s at 38 °C and 90% RH) [61]. In any case, the two polyester-based films significantly increased (*p* < 0.05) the barrier properties of paper, resulting in bilayer structures with water vapor permeances of 1.77 × 10^−10^ and 1.90 × 10^−11^ kg/m^2^·Pa·s for the paper/PBS–PBSA and paper/PET trays, respectively. These values of water vapor permeance were, respectively, two and three orders of magnitude lower than the permeance of the uncoated paper. In this regard, the water barrier enhancement attained herein is notably superior to that reported, for instance, for paper coated with PLA [48] or chitosan [62], with barrier improvements of approximately ten and five times, respectively. Therefore, both trays can be suitable for food packaging applications in humid conditions or even for storing food with high-to-moderate values of a_w_.

The transport properties of limonene vapor are also important in packaging applications because it is often used as a standard system for predicting the aroma barrier of a packaging material. As with water vapor, both polyester films offered a very noticeable improvement over the uncoated paper. In particular, the paper presented a permeance of 2.23 × 10^−9^ kg/m^2^·Pa·s, corresponding to a permeability value of 6.50 × 10^−13^ kg·m/m^2^·Pa·s [15]. Thus, the 200-µm PBS–PBSA film yielded a permeance to limonene vapor of 2.63 × 10^−10^ kg/m^2^·Pa·s, equivalent to a permeability of 5.58 × 10^−14^ kg·m/m^2^·Pa·s, whereas the 100-µm PET film showed a permeance value of 5.10 × 10^−11^ kg/m^2^·Pa·s, resulting in a permeability of 5.15 × 10^−15^ kg·m/m^2^·Pa·s (assuming a monolayer material). One can further observe that both the biopolyester blend and petrochemical polyester films offered a significant reduction (*p* < 0.05) in the aroma permeability of paper, of one and two orders of magnitude, respectively. Thus, the incorporation of the films improved the aroma barrier performance of the paper, resulting in bilayer trays of paper/PBS–PBSA and paper/PET trays with respective permeance values of 1.70 and 1.20 × 10^−10^ kg/m^2^·Pa·s. Similar to water vapor, this represents a reduction in the permeance of aroma vapor by two and three orders of magnitude, respectively, thus being also adequate to preserve aroma in food.

Oxygen barrier properties are relevant to fresh product preservation, especially when they are susceptible to oxidation processes (e.g., meat, fish, or high-lipid-content products). In the case of the uncoated sheet paper, it was not possible to determine the permeability since its permeance was above the detection limit (D.L.) of the equipment (5 × 10^−11^ m^3^/m^3.^Pa·s). The results showed, on the one hand, that the permeance of the biopolyester blend film was nearly three times higher than that of the petrochemical one, with respective values of 6.17 and 2.15 × 10^−15^ m^3^/m^2^·Pa·s. This resulted in permeabilities of 1.27 × 10^−18^ and 2.17 × 10^−19^ m^3.^m/m^2^·Pa·s, that is, one order of magnitude lower for the petrochemical polyester when considered as a monolayer material. However, it should be noted that this commercial film is based on interlayers of EVOH, a high-oxygen-barrier copolymer in low-humidity conditions (0.77 × 10^−21^ m^3.^m/m^2^·Pa·s) [61], which can be achieved in packaging structures using hydrophobic external layers (e.g., LDPE and PET) [63]. As a result, the bilayer structures presented values of permeance of 5.15 and 2.34 × 10^−15^ m^3^/m^2^·Pa·s for the paper/PBS–PBSA and paper/PET trays, respectively. Therefore, the oxygen permeance of the trays developed herein have a performance suitable for foods requiring a low or intermediate oxygen barrier. This fact, together with the high barrier to water and aroma vapors, makes the trays very suitable as packaging materials for preserving food products with high humidity but low susceptibility to oxidation.

### 3.5. Preservation of Fresh Pasta

The application of the newly developed paper-based trays in food packaging was carried out by analyzing the preservation of fresh pasta, a “fusilli calabresi” type. This food was selected due to its high a_w_ and the fact that it is locally produced in Southern Italy. Thus, it was easily accessible and is of high interest in the region. According to Italian law, “fresh pasta” can be defined as the product obtained by extrusion or lamination of a dough made of durum wheat semolina or alternative flours and water, having a moisture content >24% and a_w_ ranging between 0.92 and 0.97, whereas it requires storage at 4 ± 2 °C [64]. In this regard, the shelf life of fresh pasta depends on several factors, such as heat treatment, storage temperature, proper preservatives, and type of packaging. Industrial fresh pasta is habitually subjected to heat treatment, equivalent to pasteurization, which confers a shelf life of 30–90 days [65]. However, non-thermally treated artisanal fresh pasta has, on average, a shelf-life of only 2–3 days under refrigerated temperatures [66]. Additionally, it can be prolonged for up to 30 days with the use of preservatives and modified atmosphere packaging (MAP) [67].

**Figure 9:**
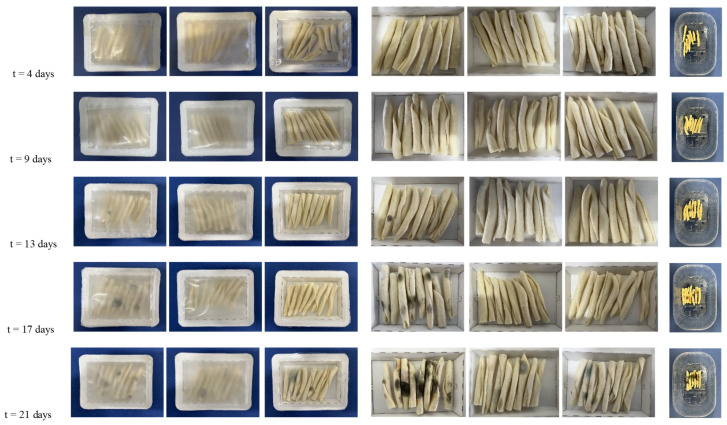
Visual images with storage time of fresh pasta packaged in trays, from left to right, of: monolayer paper, bilayer paper/poly(butylene succinate) and poly(butylene succinate-*co*-adipate) (PBS–PBSA) blend, and bilayer paper/polyethylene terephthalate (PET). (**Left pictures**): trays with the packaged pasta; (**Middle pictures**): pasta in trays after opening; (**Right pictures**): unpackaged pasta.

Shelf-life evaluation consisted of a visual observation of the surface appearance of the packaged pasta, as well as a quantification of the color parameters, weight loss, and a_w_ throughout a whole storage period of 3 weeks at 5 °C and 85% RH. This analysis was carried out in both an unopened container and the sealed trays. Figure 9 shows the visual appearance of the packaged pasta in the trays, closed and open, at the different storage times. Images of the unpackaged pasta were also taken as a reference or control to show the effect of packaging. It can be observed that, in all cases, the visual appearance of the packaged pasta was very similar during the first 4 days. However, the unpackaged pasta was slightly more yellow, suggesting loss of water and/or oxidation. After 9 days of storage, only the pasta packaged in the bilayer trays with the polyester films preserved its original brown color, while the unpackaged and packaged pasta in the monolayer paper developed a yellowish hue. For the latter samples, after 13 days of storage, visible molds were seen, which became more evident after 17 days. For the pasta stored in the paper trays with the PBS–PBSA and PET films, these acquired a yellowish hue after 13 and 17 days of storage, respectively, but both successfully harbored mold growth during this period. Lastly, after 21 days, all pasta samples presented mold overgrowth on the surface. In this regard, the spoilage of fresh pasta is generally related to mold growth [68]. Furthermore, mold spoilage is often visible to the naked eye as colonies once they reach a diameter of 3 mm [69]. For instance, cooked pasta stored in an uncoated tray was covered by approximately 80% of mold on the surface after 30 days of storage [1]. Thus, in terms of spoilage due to molds, the use of the biopolyester films on the paper substrate successfully extended the shelf life of pasta by nearly 1 week.

Fresh pasta color is a very important quality attribute since it greatly influences consumer acceptance and is the main property the consumer can evaluate when selecting a product in the market. To quantify the visual changes of the pasta during storage, the color parameters of the samples were analyzed by means of a colorimeter on their surface after opening. Thus, Table 6 shows the values of the color coordinates L*a*b* (CIELAB), where L* indicates the lightness (L* = 0 black, L* = 100 white), a* indicates the color between red (+) and green (−), and b* indicates the color between yellow (+) and blue (−). As can be observed, fresh pasta was found to have coordinates of L* = 81.3, a* = −2.2, and b* = 16.6. These color parameters are very similar to those reported by Carrini et al. [70] for fresh pasta produced using durum wheat semolina. Color evolution of the packaged pasta showed an intense reduction in brightness, decreasing from an initial L* value of 81 to values in the 77–69 range. Furthermore, the a*b* coordinates confirmed the development of a more yellowish hue, mainly due to an increase of the b* value. In particular, in terms of color variation, the unpackaged pasta showed increases in C_ab_* and h_ab_* from 16.78 and 82.57 to 21.36 and 86.40, respectively. The increase observed for the color parameter a* was previously ascribed to oxidative reactions occurring in food pasta [71]. Similarly, Zardetto and Dalla Rosa [72] indicated that, during physicochemical deterioration, fresh pasta tends to evolve to lower L* and b* parameters and higher values of a*, which are representative of a yellowish process. These color changes were mainly observed for the unpackaged pasta and, more notably, for the pasta packaged in the monolayer paper trays. Therefore, in the latter samples, although color variations were observed to occur later, the drying process was more intense. This may be ascribed to the tendency of paper to absorb moisture due to its hydrophilic nature and fibrillar structure, which could promote and favor food drying. In the case of the unpackaged pasta sample, slight but still statistically significant lower (*p* < 0.05) values than in the paper-packaged pasta were observed. This fact can be related to the high humidity of the chamber (∼85%), although color changes occurred faster. Lastly, it was confirmed that the pasta packaged in the bilayer paper/PBS–PBSA and paper/PET trays nearly maintained the original color for 13 and 17 days, respectively.

**Figure 10:**
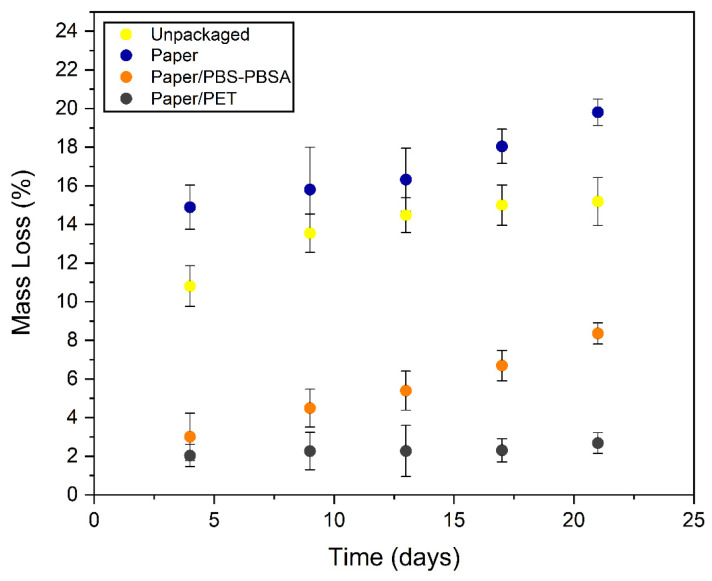
Percentage of mass loss of unpackaged and packaged fresh pasta in trays of monolayer paper, bilayer paper/poly(butylene succinate) and poly(butylene succinate-*co*-adipate) (PBS–PBSA) blend, and bilayer paper/polyethylene terephthalate (PET).

The initial moisture content of the fresh pasta was approximately 32 g of water per 100 g of product. Then, the weight changes that occurred in the different packaged pasta samples over time were monitored. Figure 10, which shows the evolution of mass loss with storage time, confirmed that a drying process occurred in the fresh pasta since the samples showed a continuous increase in mass loss over time. Mass loss evolution also confirmed that this drying process was more intense in the unpackaged and paper-packaged pasta samples. As previously suggested during the color analysis, the unpackaged pasta samples showed lower mass loss due to drying than the pasta packaged in the monolayer paper trays, but the mass of these samples stabilized faster, after 13 days of storage. However, all the food samples packaged in the paper trays, both monolayer and bilayers, showed a continuous and progressive loss of mass during the whole storage period. It can be observed that all mass losses were significantly different (*p* < 0.05) among the pasta samples packaged in the different paper trays, which correlated well with the water barrier properties determined above for each material. Thus, the lowest mass loss was attained in the case of the pasta packaged in the paper/PET trays, reaching values of 2.7% after 3 weeks of storage, whereas the food samples packaged in the paper/PBS–PBSA trays yielded values of 8.4%. However, compared to the monolayer paper tray sample, in which the mass loss reached a value of nearly 20%, the mass loss in the bilayer trays with the biopolymer blend was approximately three times lower. In this context, Sousa et al. [72] reported that food pastas intercalated with biodegradable films made from rice flour, PBAT, and glycerol containing different amounts of potassium sorbate suffered a gradual reduction in moisture content, ranging from 16% to 28%, after 2 weeks of storage. This result confirms the relatively good protection that the biopolyester film can offer to the paper trays against moisture, with a notable capacity to reduce the drying process in the food, particularly during the first 13 days of storage.

**Figure 11:**
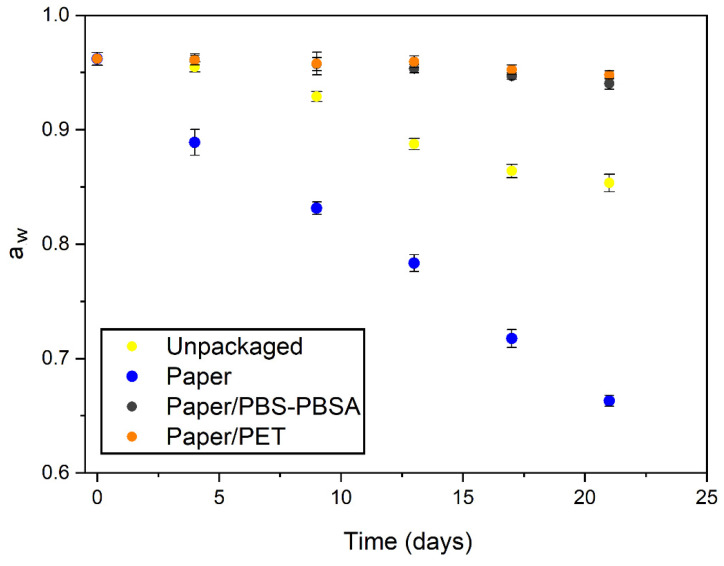
Evolution of water activity (a_w_) of unpackaged and packaged fresh pasta in trays of monolayer paper, bilayer paper/poly(butylene succinate) and poly(butylene succinate-*co*-adipate) (PBS–PBSA) blend, and bilayer paper/polyethylene terephthalate (PET).

To conclude the shelf-life analysis, the a_w_ of the pasta packaged in the paper trays was evaluated as function of the storage time. This physicochemical property refers to the free and available water content in the food. It is, therefore, a very useful parameter for determining the drying process extension and, consequently, the loss of quality in humid foods [73]. Moreover, a_w_ has been shown to be a determinant factor for the growth of microorganisms and is well related to most degradation reactions of chemical, enzymatic, and physical nature observed in pasta [74]. As shown in Figure 11, the as-received fresh pasta presented a value of a_w_ of 0.962, which provides sufficient moisture to support the growth of bacteria, yeasts, and mold, thus making this product vulnerable to fast spoilage [75]. This value was very close to the legal limit, 0.97 [76], but it must be taken into consideration that this pasta did not undergo pasteurization, which is known to slightly reduce a_w_. Very similar a_w_ values, in the 0.97–0.95 range, have been reported for Italian fresh pasta [77]. It can be observed that a_w_ was sharply reduced during storage in the fresh pasta packaged in the monolayer paper trays, reaching a value as low as 0.663 after 3 weeks. The reduction in a_w_ in the unpackaged pasta was significantly lower (*p* < 0.05), which also stabilized to a value of 0.854 after 3 weeks due to the fact that the sample was able to reach equilibrium with the relative humidity of the chamber (~85%). It is worth noting that a_w_ values of 0.8 or even lower can support the growth of molds, which would explain the presence of these organisms in some of the dried food samples. For instance, Xiong et al. [78] showed that the decrease in a_w_ from approximately 0.955 to below 0.925 by applying thermal treatments reduced microbial growth in fresh noodles, but other factors such as secondary contamination and air exposure also had adverse effects on the quality during storage. Furthermore, the a_w_ values for the pasta samples packaged in the bilayer paper/PBS–PBSA and paper/PET trays were very similar, reaching values in the 0.94–0.95 range after 3 weeks of storage, being slightly lower for the biopolyester-containing structure but, interestingly, with no significant differences (*p* > 0.05). In this regard, Sanguinetti et al. [79], who studied the evolution of a_w_ with storage time, showed that MAP packaging can keep gluten-free fresh pasta at the 0.96–0.95 range for 42 days. The latter result, which was nearly in the same range of a_w_, confirms the high potential of the newly developed trays of paper and bio-based polyesters for preserving fresh pasta.

### 3.6. Overall Migration

The migration of the packaging constituents into food is an important issue in food contact materials from the point of view of food safety. However, it has scarcely been investigated in biopolymers developed for food packaging applications. Thus, ethanol 10% *v*/*v* (simulant A) and Tenax (simulant E) were chosen to simulate migration into fresh pasta via direct contact, without immersion, at 40 °C for 10 days. Food simulant A was chosen due to the high a_w_ of fresh pasta, whereas the selection of simulant E was based on the fact that it is habitually employed for paper-based packaging analysis due to the poor moisture resistance of paper. The results, shown in Table 7, indicate that all tested materials successfully complied with the overall migration limit (OML) set in Commission Regulation (EU) No. 10/2011 [21] on plastic materials and articles intended to come into contact with food. This is also valid for its subsequent amendment for each tested simulant under the evaluated exposure conditions. In particular, for both tests, the regulation sets a threshold value of 10 mg/dm^2^ (maximum OML as sum of all substances that can migrate from the food contact material to the food simulant). In the case of the simulant A, the biopolyester blend film yielded a migration value of 3.6 mg/dm^2^, whereas the bilayer tray showed a significantly lower (*p* < 0.05) value, equal to 1.9 mg/dm^2^. Since, in both cases, the food simulant A was in contact with the PBS–PBSA layer, the lower value observed in the bilayer tray sample can be ascribed to the lamination process of the film onto paper during thermoforming or potential absorption of hydrophilic substances in the paper layer. Similar results were recently attained for films of PHBV, a microbial copolyester, showing values in the 1–3 mg/dm^2^ range [80]. In the case of the food simulant E, both samples yielded values lower than 2 mg/dm^2^, showing no significant differences (*p* > 0.05) and being well below the legal threshold. Therefore, it can be concluded that the newly developed trays can be safely applied to preserve fresh pasta and other types of foods with high a_w_. Furthermore, according to the legislation, the particular conditions selected herein also cover the packaging material being in contact for longer periods at room temperature or lower temperatures. Lastly, they also cover high-temperature conditions and/or packaging subjected to heating processes (from 70 °C for 2 h up to 100 °C for 15 min).

## 4. Conclusions

Lamination of renewable succinic acid derived polyester blend films on paper substrate by thermoforming was demonstrated herein to be a very effective strategy for developing sustainable trays with improved thermal, mechanical, and barrier properties. The physical properties of the newly developed paper/PBS–PBSA were slightly lower but in the same range of paper bilayer structures developed via the same process using PET-based high-barrier multilayer films. In particular, the incorporation of the PBS–PBSA films nearly doubled the elongation during flexural analysis, whereas the puncture resistance was increased approximately fivefold. Moreover, it successfully improved the moisture and aroma vapor resistance by two orders of magnitude and endowed paper with intermediate oxygen gas barrier properties, reaching values of permeability in the range of medium-barrier plastic materials. These novel paper/PBS–PBSA trays were successfully applied, for the first time, to package fresh pasta, a traditional Italian food with high a_w_, where the use of uncoated paper is currently restricted. Shelf-life evaluation carried out under refrigerated conditions (5 °C and 85% HR) for a period of 21 days determined that the application of the biopolyester blend film extended the physicochemical properties of the packaged fresh pasta for approximately 1 week. In particular, the biopolymer blend film delayed color changes and mold growth, as well as contributed to reducing the drying process of fresh pasta. Accordingly, the results of this physicochemical quality analysis suggest acceptance of the pasta packaged in paper/PBS–PBSA trays within 9 days of storage. Moreover, overall migration studies carried out with two different simulants showed that the newly developed paper/PBS–PBSA trays comply with current legislation on plastic materials and articles intended to come into contact with food. Therefore, these paper/biopolyester bilayer trays can be regarded as excellent candidates for food packaging applications since these are based on renewable and biodegradable materials, and they can be organically composted after use. These packaging materials can replace, for instance, current paper structures based on petrochemical polymer films, without compromising the renewability, recyclability, and compostability of paper.

## Figures and Tables

**Table 1:** Color parameters (L*, a*, b*), color saturation or chroma (C_ab_*), hue angle (h_ab_*), and color difference (∆E_ab_*) of the paper sheet, poly(butylene succinate) and poly(butylene succinate-*co*-adipate) (PBS–PBSA) blend and polyethylene terephthalate (PET) films, and paper/PBS–PBSA and paper/PET trays.

Sample	L*	a*	b*	C_ab_*	h_ab_*	ΔE_ab_*
Paper	94.77 ± 0.01 ^a^	1.56 ± 0.02 ^a^	−5.83 ± 0.06 ^a^	6.03 ± 0.07 ^a^	284.94 ± 0.04 ^a^	-
PBS–PBSA	89.41 ± 0.88 ^b^	−0.44 ± 0.04 ^b^	1.44 ± 0.48 ^b^	1.52 ± 0.44 ^b^	108.44 ± 8.17 ^b^	-
PET	88.03 ± 0.36 ^c^	−1.54 ± 0.06 ^c^	4.25 ± 0.11 ^c^	4.52 ± 0.12 ^c^	108.89 ± 0.45 ^b^	-
Paper/PBS–PBSA	94.45 ± 0.15 ^d^	0.22 ± 0.05 ^d^	−0.59 ± 0.06 ^d^	0.63 ± 0.07 ^d^	290.43 ± 2.07 ^c^	5.42 ± 0.10 ^a^
Paper/PET	95.13 ± 0.21 ^e^	0.84 ± 0.01 ^e^	−3.48 ± 0.05 ^e^	3.58 ± 0.05 ^e^	283.57 ± 1.81 ^a^	2.48 ± 0.09 ^b^

Different superscript letters (a–e) indicate significant differences among formulations (*p* < 0.05).

**Table 2:** Thermal stability in terms of degradation onset temperature (T_onset_), degradation temperature (T_deg_), and remaining mass at 700 °C of the paper sheet, poly(butylene succinate) and poly(butylene succinate-*co*-adipate) (PBS–PBSA) blend and polyethylene terephthalate (PET) films, and paper/PBS–PBSA and paper/PET trays.

Sample	T_onset_ (*°*C)	T_deg_ (*°*C)			Remaining Mass (%)
		T_deg 1_	T_deg 2_	T_deg 3_	
Paper	279.6 ± 5.3 ^a^	335.1 ± 0.1 ^a^	472.5 ± 4.7 ^a^	-	16.3 ± 0.12 ^a^
PBS–PBSA	297.5 ± 7.8 ^b^	392.5 ± 0.5 ^b^	-	-	0.3 ± 0.4 ^b^
PET	298.0 ± 2.8 ^b^	353.5 ± 0.7 ^c^	434.3 ± 4.1 ^b^	544.0 ± 1.4 ^a^	0.5 ± 0.5 ^b^
Paper/PBS–PBSA	284.5 ± 3.5 ^a^	381.5 ± 3.1 ^d^	479.2 ± 1.9 ^c^	-	6.0 ± 1.6 ^c^
Paper/PET	287.5 ± 3.5 ^ab^	336.2 ± 0.5 ^e^	429.0 ± 4.2 ^b^	491.5 ± 14.8 ^b^	6.5 ± 1.2 ^c^

Different superscript letters (a–e) indicate significant differences among formulations (*p* < 0.05).

**Table 3:** Tensile properties in terms of tensile modulus (E_tensile_), stress at yield (σ_y_), and percentage elongation at break (%ε_b_) of the paper sheet, poly(butylene succinate) and poly(butylene succinate-*co*-adipate) (PBS–PBSA) blend and polyethylene terephthalate (PET) films, and paper/PBS–PBSA and paper/PET trays prior and after 21 days of storage (5 °C and 85% RH).

Sample	Initial Day			21 Days		
	E_tensile_ (MPa)	σ_y_ (MPa)	ε_b_ (%)	E (MPa)	σ_y_ (MPa)	ε_b_ (%)
Paper	1787 ± 41 ^a,1^	31.9 ± 1.0 ^a,1^	6.9 ± 0.9 ^a,1^	1124 ± 78 ^a,2^	41.7 ± 3.3 ^a,2^	9.2 ± 0.0 ^a,2^
PBS–PBSA	367 ± 15 ^b,1^	21.6 ± 1.1 ^b,1^	158.4 ± 26.5 ^b,1^	359 ± 14 ^b,1^	23.2 ± 1.3 ^b,1^	156.3 ± 10.9 ^b,1^
PET	263 ± 31 ^c,1^	8.8 ± 0.2 ^c,1^	303.9 ± 82.4 ^c,1^	267 ± 21 ^c,1^	12.9 ± 0.6 ^c,2^	331.2 ± 28.5 ^c,1^
Paper/PBS–PBSA	1081 ± 31 ^d,1^	49.6 ± 2.2 ^d,1^	6.6 ± 0.5 ^a,1^	859 ± 34 ^d,2^	36.5 ± 0.9 ^d,2^	11.2 ± 0.7 ^d,2^
Paper/PET	946 ± 22 ^e,1^	32.3 ± 1.4 ^a,1^	8.9 ± 0.4 ^d,1^	924 ± 38 ^d,1^	32.6 ± 2.8 ^e,1^	8.4 ± 0.7 ^e,1^

Different superscript letters (a–e) indicate significant differences among formulations for the same storage time and different superscript numbers indicate differences due to storage time for the same sample (1–2) (*p* < 0.05).

**Table 4:** Flexural properties in terms of elastic modulus (E _flexural_), stress at yield (σ_y flexural_), and percentage elongation at yield (%ε_y flexural_) and puncture resistance in terms of maximum force (F_max_), total displacement (d_total_), and total energy (E_puncture_) of the paper sheet and paper/poly(butylene succinate) and poly(butylene succinate-*co*-adipate) (PBS–PBSA) blend and paper/polyethylene terephthalate (PET) trays.

Sample	Flexural Test			Puncture Test		
	E_flexural_ (MPa)	σ_y flexural_ (MPa)	ε_y flexural_ (%)	F_max_ (N)	d_total_ (mm)	E_puncture_ (mJ)
Paper	1498 ± 34 ^a^	30.4 ± 2.1 ^a^	0.69 ± 0.1 ^a^	53 ± 4 ^a^	1.4 ± 0.1 ^a^	25 ± 2 ^a^
Paper/PBS–PBSA	1235 ± 23 ^b^	35.9 ± 1.3 ^b^	1.14 ± 0.2 ^b^	64 ± 5 ^b^	5.4 ± 0.5 ^b^	130 ± 7 ^b^
Paper/PET	912 ± 32 ^c^	20.3 ± 0.9 ^c^	1.36 ± 0.2 ^b^	56 ± 3 ^ab^	5.3 ± 0.6 ^b^	85 ± 4 ^c^

Different superscript letters (a–c) indicate significant differences among formulations (*p* < 0.05).

**Table 5:** Permeance and permeability to water and _D_-limonene vapors and oxygen of the paper sheet, poly(butylene succinate) and poly(butylene succinate-*co*-adipate) (PBS–PBSA) blend and polyethylene terephthalate (PET) films, and paper/PBS–PBSA and paper/PET trays.

Sample	Thickness	Water Vapor		Limonene Vapor		Oxygen Gas	
	(µm)	Permeance (kg/Pa·s·m^2^)	Permeability (kg·m/Pa·s·m^2^)	Permeance (kg/Pa·s·m^2^)	Permeability (kg·m/Pa·s·m^2^)	Permeance (m^3^/m·s·Pa)	Permeability (m^3^·m/m^2^·s·Pa)
		×10^10^	×10^15^	×10^10^	×10^15^	×10^15^	×10^19^
Paper	291 ± 6 ^a^	110.16 ± 8.89 ^a^	3205.65 ± 87.11 ^a^	22.34 ± 1.34 ^a^	650.15 ± 9.42 ^a^	>D.L.	>D.L.
PBS–PBSA	212 ± 5 ^b^	1.47 ± 0.07 ^b^	31.56 ± 0.96 ^b^	2.63 ± 0.08 ^b^	55.76 ± 2.66 ^b^	6.17 ± 0.14 ^a^	12.74 ± 0.30 ^a^
PET *	101 ± 1 ^c^	0.55 ± 0.07 ^c^	5.58 ± 0.71 ^c^	0.51 ± 0.02 ^c^	5.15 ± 0.19 ^c^	2.15 ± 0.01 ^b^	2.17 ± 0.02 ^b^
Paper/PBS–PBSA	461 ± 19 ^d^	1.77 ± 0.77 ^b^	-	1.70 ± 0.14 ^d^	-	5.15 ± 0.02 ^c^	-
Paper/PET	350 ± 3 ^e^	0.19 ± 0.01 ^d^	-	1.20 ± 0.22 ^e^	-	2.34 ± 0.01 ^d^	-

* Assuming a monolayer material. Different superscript letters (a–e) indicate significant differences among formulations (*p* < 0.05).

**Table 6:** Color parameters (L*, a*, b*), color saturation or chroma (C_ab_*), hue angle (h_ab_*), and color difference (∆E_ab_*) of unpackaged and packaged fresh pasta in trays of monolayer paper, bilayer paper/poly(butylene succinate) and poly(butylene succinate-*co*-adipate) (PBS–PBSA) blend, and bilayer paper/polyethylene terephthalate (PET).

Time (Days)	Sample	L*	a*	b*	C_ab_*	h_ab_*	ΔE_ab_*
0	Initial	81.33 ± 1.24 ^1^	−2.17 ± 0.36 ^1^	16.64 ± 1.78 ^1^	16.78 ± 1.58 ^1^	82.57 ± 2.85 ^1^	-
4	Unpackaged	75.47 ± 1.74 ^a,2^	−2.01 ± 0.76 ^a,1^	19.36 ± 2.02 ^a,12^	19.46 ± 1.56 ^a,2^	84.07 ± 2.18 ^a,1^	6.46 ± 1.08 ^c,1^
	Paper	78.98 ± 1.10 ^b,2^	−2.10 ± 0.55 ^a,1^	18.36 ± 1.87 ^a,12^	18.48 ± 1.24 ^a,2^	83.47 ± 2.78 ^a,1^	2.91 ± 0.47 ^b,1^
	Paper/PBS–PBSA	79.89 ± 1.83 ^b,12^	−2.36 ± 0.56 ^a,1^	17.30 ± 1.16 ^a,1^	17.46 ± 1.23 ^a,1^	82.23 ± 1.98 ^a,1^	1.60 ± 0.28 ^a,1^
	Paper/PET	79.93 ± 1.02 ^b,12^	−2.72 ± 0.98 ^a,1^	16.81 ± 1.34 ^a,1^	17.03 ± 1.39 ^a,1^	80.81 ± 2.13 ^ab,1^	1.51 ± 0.34 ^a,1^
9	Unpackaged	74.07 ± 1.45 ^a,2^	−1.98 ± 0.89 ^a,1^	20.96 ± 1.23 ^a,2^	21.05 ± 1.45 ^a,3^	84.60 ± 2.45 ^a,2^	8.45 ± 1.35 ^c,2^
	Paper	77.52 ± 1.52 ^b,2^	−2.65 ± 0.72 ^a,1^	20.06 ± 1.63 ^a,2^	20.23 ± 1.03 ^a,3^	82.47 ± 2.12 ^b,2^	5.14 ± 1.06 ^b,2^
	Paper/PBS–PBSA	78.08 ± 1.10 ^b,23^	−1.97 ± 0.89 ^a,1^	18.24 ± 1.68 ^a,1^	18.35 ± 1.06 ^b,2^	83.84 ± 2.05 ^b,2^	3.63 ± 0.57 ^a,2^
	Paper/PET	78.55 ± 1.23 ^b,23^	−2.41 ± 0.45 ^a,1^	18.32 ± 1.56 ^a,1^	18.48 ± 1.16 ^b,2^	82.51 ± 1.89 ^b,2^	3.26 ± 0.48 ^a,2^
13	Unpackaged	73.87 ± 1.69 ^a,2^	−2.18 ± 0.34 ^a,1^	21.34 ± 1.26 ^a,2^	21.45 ± 1.24 ^a,3^	84.17 ± 1.98 ^a,2^	8.82 ± 1.59 ^d,2^
	Paper	75.63 ± 2.45 ^ab,23^	−2.98 ± 0.65 ^a,1^	21.72 ± 2.09 ^a,2^	21.92 ± 1.20 ^a,3^	82.19 ± 2.38 ^b,2^	7.68 ± 1.32 ^c,3^
	Paper/PBS–PBSA	76.64 ± 2.39 ^ab,2345^	−2.12 ± 0.87 ^a,1^	19.58 ± 1.34 ^a,1^	19.69 ± 1.38 ^b,2^	83.82 ± 2.57 ^b,2^	5.54 ± 1.02 ^b,3^
	Paper/PET	78.42 ± 1.45 ^b,23^	−2.57 ± 0.69 ^a,1^	18.93 ± 1.07 ^b,1^	19.10 ± 1.56 ^b,2^	82.27 ± 3.04 ^b,2^	3.72 ± 0.67 ^a,3^
17	Unpackaged	73.02 ± 1.57 ^a,2^	−1.98 ± 0.49 ^a,1^	21.16 ± 2.46 ^a,2^	21.25 ± 1.86 ^a,3^	84.65 ± 2.08 ^a,2^	9.46 ± 1.77 ^c,3^
	Paper	71.04 ± 3.21 ^a,34^	−3.01 ± 0.89 ^a,1^	21.02 ± 3.11 ^a,2^	21.23 ± 1.32 ^a,3^	81.85 ± 3.19 ^b,2^	11.21 ± 1.65 ^c,4^
	Paper/PBS–PBSA	75.34 ± 1.70 ^a,345^	−2.56 ± 0.65 ^a,1^	18.91 ± 1.78 ^a,1^	19.08 ± 1.68 ^b,2^	82.29 ± 2.75 ^b,2^	6.42 ± 1.21 ^b,4^
	Paper/PET	78.19 ± 0.78 ^b,23^	−2.29 ± 0.85 ^a,1^	18.89 ± 1.63 ^a,1^	19.03 ± 1.92 ^b,2^	83.09 ± 2.63 ^b,2^	3.86 ± 0.46 ^a,4^
21	Unpackaged	72.27 ± 2.86 ^a,2^	−1.34 ± 0.90 ^a,1^	21.32 ± 2.36 ^a,2^	21.36 ± 1.65 ^a,3^	86.40 ± 1.88 ^a,3^	10.23 ± 1.98 ^c,4^
	Paper	69.07 ± 2.86 ^a,4^	−1.58 ± 0.87 ^a,1^	21.26 ± 2.65 ^a,2^	21.32± 1.76 ^a,3^	85.75 ± 2.14 ^a,3^	13.11 ± 1.87 ^c,4^
	Paper/PBS–PBSA	73.04 ± 1.66 ^a,5^	−2.77 ± 0.56 ^a,1^	20.91 ± 1.12 ^a,1^	21.09± 1.37 ^a,3^	82.45 ± 2.67 ^b,3^	9.34 ± 1.78 ^b,5^
	Paper/PET	76.89 ± 0.92 ^b,3^	−2.68 ± 0.71 ^a,1^	19.97 ± 1.85 ^a,1^	20.15± 1.62 ^a,3^	82.36 ± 2.73 ^b,3^	5.57 ± 1.02 ^a,5^

Different superscript letters (a–d) indicate significant differences among formulations (*p* < 0.05). Different superscript numbers (1–5) indicate significant differences between the storage times (*p* < 0.05).

**Table 7:** Overall migration levels of monolayer paper sheet, poly(butylene succinate) and poly(butylene succinate-*co*-adipate) (PBS–PBSA) blend film, and bilayer paper/PBS–PBSA tray into food simulants.

Sample	Ethanol 10% *v*/*v* (mg/dm^2^)	Tenax (mg/dm^2^)
Paper	-	1.6 ± 0.2 ^a^
PBS–PBSA	3.6 ± 0.3 ^a^	-
Paper/PBS–PBSA	1.9 ± 0.2 ^b^	1.5 ± 0.1 ^a^

Different superscripts within the same column indicate significant differences among samples (a,b) (*p* < 0.05).

## Data Availability

Data are contained within the article and are also available on request.

## Supplementary Materials

The following supporting information can be downloaded at https://www.mdpi.com/article/10.3390/ma16103872/s1: Video S1. Video showing a representative test for seal strength evaluation of paper/PBS–PBSA in the universal testing machine; Video S2. Video showing a representative test for seal strength evaluation of paper/PET in the universal testing machine.
